# Long noncoding RNA UCA1 as a novel biomarker of lymph node metastasis and prognosis in human cancer: a meta-analysis

**DOI:** 10.1042/BSR20180995

**Published:** 2019-04-26

**Authors:** Congmin Liu, Jing Jin, Jin Shi, Liqun Wang, Zhaoyu Gao, Tiantian Guo, Yutong He

**Affiliations:** Cancer Institute, The Fourth Hospital of Hebei Medical University/The Tumor Hospital of Hebei Province, Shijiazhuang, Hebei 050000, China

**Keywords:** cancer, lncRNA, lymph node metastasis, prognosis meta-analysis, UCA1

## Abstract

**Background:** Urothelial carcinoma associated 1 (UCA1), a novel long noncoding RNA (lncRNA) which is first discovered in 2006 in human bladder cancer and has become a hot spot in recent years. UCA1 has been demonstrated correlated with clinical outcomes in various cancers. However, the results from each study are insufficient and not completely consistent. Therefore, we perform a systematic meta-analysis to evaluate the value for a feasible biomarker for metastasis and prognosis of cancer. **Methods:** Relevant English literatures were searched in PubMed, Cochrane Library, Web of science, Embase databases and Chinese literatures were searched in Chinese National Knowledge Infrastructure Wanfang from inception up to 17 April 2018. The pooled odds ratio (OR) and hazard ratio (HR) with 95% confidence interval (CI) using random/fixed-effect were used to identify the relationship between UCA1 and lymph node metastasis (LNM) or overall survival (OS) of cancer patients. Subgroup analysis and sensitivity analysis were performed. The current meta-analysis was performed using Review Manager 5.3 and Stata 12.0 software. **Results:** A total of 3411 patients from 38 studies were finally included. Patients who with high UCA1 expression suffered from an increased risk of LNM (OR = 2.50; 95% CI: 1.93–3.25). UCA1 was also significantly associated with OS (HR = 2.05; 95% CI: 1.77–2.38). Subgroup analyses across several different variables also showed the similar results in LNM and OS of cancer patients. **Conclusion:** High expression of UCA1 was linked with poor clinical outcome. UCA1 can serve as a potential molecular marker for metastasis and prognosis in different types of cancers.

## Introduction

Cancer is a major global public health problem that seriously threatens human health. In recent years, the incidence and mortality of cancer are also increasing year by year. According to GLOBCAN 2012, there were 14.1 million new cancer cases, 8.2 million cancer deaths and 32.6 million people living with cancer (within 5 years of diagnosis) in 2012 worldwide [1]. In the United States, cancer is the second leading cause of death with an estimated 1,685,210 new cases and 595,690 deaths cancer in 2016 [2]. In China, cancer has been the leading cause of death with an estimated 4,292,000 new cases and 2,814,000 death cases in 2015 [3]. The current strategies to cancer therapy have significantly improved in some types of cancer, such as surgery, radiotherapy or chemotherapy. However, the outcome still remains undesirable. Therefore, looking for effective molecular biomarkers which can be used to evaluate potential risk of cancer is becoming imminent.

With the development of second-generation sequencing technology, more and more long noncoding RNAs (lncRNAs) was been found. LncRNAs were defined as non-protein coding RNAs with the length of more than 200 nucleotides. Recent studies have shown that lncRNAs are closely associated with diverse biological processes, especially in various types of cancer and played an indispensable role in the metastasis and prognosis of cancer [4]. Noteworthily, lncRNAs either could be acted as oncogenes or tumor suppressors in multiple cancers, such as HOPPIP [5] and MEG3 [6]. Urothelial carcinoma associated 1 (UCA1), also known as cancer-resistant drug resistance gene, a 2314-bp lncRNA encoded on human chromosome 19p13.12. UCA1 was a novel lncRNA which was first discovered in 2006 in human bladder cancer and has become a hot spot in recent years [7,8]. Accumulating evidence revealed that UCA1 was dysregulated in cancer tissues and participated in the malignant progression of cancers, including bladder cancer, breast cancer, gastric cancer (GC), colorectal cancer (CRC) and lung cancer [9]. Studies have shown that the dysregulation of UCA1 is closely associated with the clinicopathological characteristics of cancer, such as lymph node metastasis (LNM) and overall survival (OS). However, since the results of the studies were not consistent and small sample size in individual study, we collected relevant publications and performed a meta-analysis to investigate the relationship between UCA1 expression and lymph node metastasis or prognosis, aiming to further evaluate whether the UCA1 could be served as a potential molecular biomarker for cancers.

## Materials and methods

### Literature collection

We searched the electronic databases PubMed, Cochrane Library, Web of science, Embase databases, Chinese National Knowledge Infrastructure (CNKI) and Wanfang, by using ‘UCA1 or urothelial carcinoma associated 1’ as the keywords, in order to obtain potential articles referenced in the publications. Retrieval time for the last update is up to 17 April 2018.

### Inclusion and exclusion criteria

Inclusion criteria for the articles were as the following: (1) Evaluation of the relationship between UCA1 expression and metastasis, or prognosis of patients in human cancer. (2) Patients were divided into high and low expression group according to the expression levels of UCA1. (3) Related clinicopathologic parameters and outcomes were described, such as LNM and OS. (4) Sufficient data for calculating odds ratio (OR), hazard ratio (HR) and its corresponding 95% confidence intervals (CI).

Exclusion criteria for the articles were as follows: (1) Nonhuman research, reviews, editorials, expert opinions, letters and case reports. (2) Duplicate publications. (3) Studies without valuable data.

### Date extraction

Two investigators (H.Y.T. and L.C.M.) extracted and reviewed the essential data from the included studies independently, according to the inclusion and exclusion criteria. Disagreements were solved by two investigators (J.J. and S.J.) by discussions. For each eligible study, we extracted the following information: first author, publication year, tumor type, country, total number of patients, detection method of UCA1, UCA1 expression levels, number of high UCA1 expression group and low UCA1 expression group, number of patients with LNM, follow-up duration, reference control, HRs as well as their 95% CIs.

### Quality assessment

The quality of all included studies was assessed by two investigators (W.L.Q. and G.Z.Y.) according to the Newcastle–Ottawa Scale (NOS) independently. For any divergence, a consensus was reached by a third investigator (GTT). NOS scores ranged from 0 to 9 points, with higher scores indicated a better quality and all included eligible studies were assessed to be of high quality by using the NOS in this meta-analysis.

### Statistical analysis

The association between UCA1 and cancer lymph node metastasis or prognosis was assessed by OR and HR with its corresponding 95% CI. The current meta-analysis was performed through Review Manager 5.3 and Stata 12.0 software. We use the χ^2^-based Q test and I^2^ statistics evaluate the heterogeneity of the eligible studies. The random-effects model was used to analyze the results when heterogeneity was present (*I^2^* > 50%, *P*<0.05); while the fixed-effects model was applied for this meta-analysis when the heterogeneity was absent in eligible studies (*I^2^* < 50%, *P*>0.05). The potential publication bias was assessed with the Begg’s funnel-plot. The *P*-value less than 0.05 was considered to be statistically significant.

## Results

**Figure 1:**
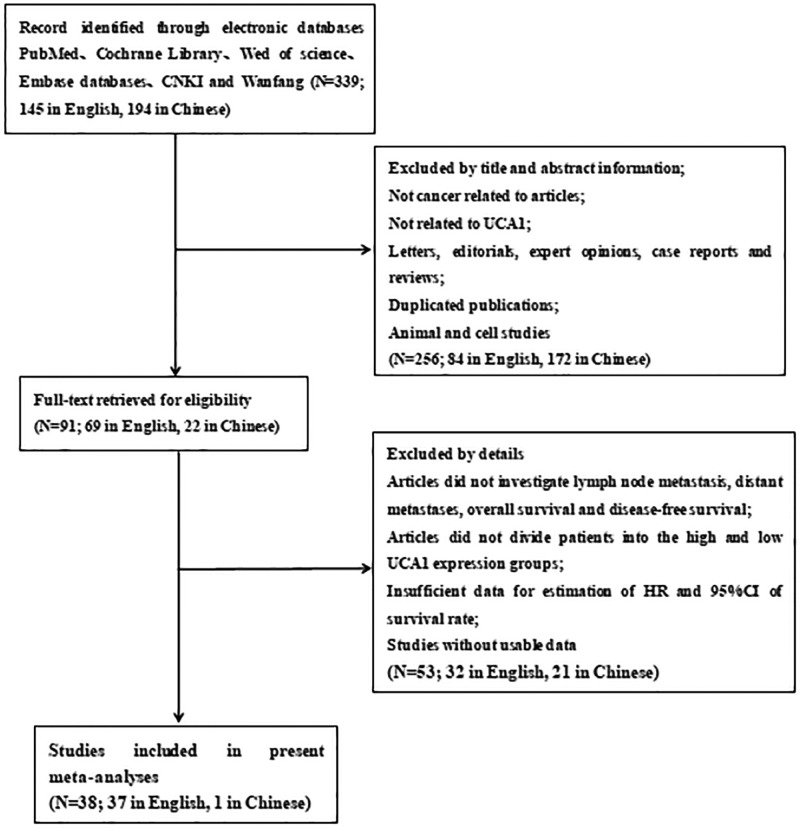
The flow diagram of the meta-analysis

As shown in Figure 1, a total of 339 published articles were identified from the first attempt to search by using the keywords, of which 145 in English and 194 in Chinese. After removing duplicates, then screening the title and abstract carefully, 248 articles were excluded. After further inspection of the full articles, 53 articles were excluded. Eventually, according to the criteria for selection, a total of 38 studies, of which 1 is in Chinese and the others are in English, were included in the current meta-analysis.

## Literature search and study characteristics

Tables 1 and 2 showed the main characteristics of the included researches. A total of 38 studies [10–47] involving 3411 cancer patients were included. The average patient sample size is 89.76, the maximum sample size is 384, and the minimum sample size is 20. Among the 38 studies, UCA1 was tested in 19 types of cancers, six studies focused on GC, four studies focused on CRC, three studies concentrated on prostate cancer and hepatocellular carcinoma (HCC), respectively; two study on renal cell carcinoma (RCC), ovarian cancer, (OC) pancreatic ductal adenocarcinoma (PDAC) and glioma, respectively, one study on non-small-cell lung cancer (NSCLC), lung cancer, esophageal cancer, esophageal squamous cell carcinoma, gallbladder cancer (GBC), oral squamous cell carcinoma, hypopharyngeal squamous cell carcinoma, urothelial carcinoma (UC), pancreatic cancer, endometrial cancer, breast cancer, osteosarcoma, colon cancer, cholangiocarcinoma (CCA) and multiple myeloma (MM), respectively. All the diagnoses of LNM were based on pathology. In all of the included studies, the patients were divided into two groups: high and low expression of UCA1. All studies used quantitative real-time PCR (qRT-PCR) to detect the expression of UCA1. The main characteristics of the eligible studies were summarized in Tables 1 and 2.

**Table 1:** Characteristics of studies about prognosis in this meta-analysis

Author	Year	Tumor type	Country	Sample size	UCA1 assay	Reference controls	UCA1 expression				Cut-off value	Research type of the studies
							High expression	High with LNM	Low expression	Low with LNM		
Bian	2016	CRC	China	90	qRT-PCR	β-Actin	45	30	45	23	Median	Case–control study
Cai Q.	2017	GBC	China	45	qRT-PCR	GAPDH	23	12	22	18	Median	Case–control study
Chen P.	2016	Pancreatic	China	128	qRT-PCR	GAPDH	64	42	64	32	Median	Case–control study
Fu	2016	PDAC	China	80	qRT-PCR	GAPDH	40	17	40	17	Median	Case–control study
Han	2014	CRC	China	80	qRT-PCR	GAPDH	37	17	43	18	Mean	Case–control study
He	2017	Glioma	China	80	qRT-PCR	β-Actin	51	28	29	8	NA	Case–control study
Khakiani	2017	GC	Iran	40	qRT-PCR	GUSB	20	9	20	5	Median	Case–control study
Li	2014	ESCC	China	90	qRT-PCR	GAPDH	41	22	49	12	Mean	Case–control study
Li L.	2017	GC	China	102	qRT-PCR	GAPDH	73	44	29	10	NA	Case–control study
Lu	2016	EC	China	45	qRT-PCR	GAPDH	12	7	33	5	Median	Case–control study
Ni	2015	CRC	China	54	qRT-PCR	GAPDH	27	12	27	5	Median	Case–control study
Nie	2016	NSCLC	China	112	qRT-PCR	β-Actin	39	14	73	21	Youden index	Case–control study
Qian	2017	HCC	China	53	qRT-PCR	β-Actin	26	17	27	9	Median	Case–control study
Tao	2015	CRC	China	80	qRT-PCR	β-Actin	20	13	60	21	Fourth quartile of the expression of UCA1	Case–control study
Wang F.	2015	HCC	China	98	qRT-PCR	RNU6B	49	30	49	11	Median	Case–control study
Wang H.	2015	LC	China	60	qRT-PCR	GAPDH	36	26	24	8	Median	Case–control study
Wang Z.	2017	GC	China	39	qRT-PCR	GAPDH	22	18	17	7	Relative expression ratios <0.5	Case–control study
Wen	2017	Osteosarcoma	China	151	qRT-PCR	GAPDH	75	44	76	28	NA	Case–control study
Xu	2017	CCA	China	68	qRT-PCR	GAPDH	38	26	30	12	NA	Case–control study
Yang Y.J.	2016	OC	China	53	qRT-PCR	GAPDH	27	13	26	5	Median	Case–control study
Yang Y.T.	2016	OSCC	China	124	qRT-PCR	GAPDH	62	35	62	20	NA	Case–control study
Zhang L.	2016	OC	China	110	qRT-PCR	GAPDH	57	26	53	12	Median	Case–control study
Zheng	2015	GC	China	112	qRT-PCR	RNU6B	56	35	56	37	Median	Case–control study
Zhou	2017	PC	China	72	qRT-PCR	GAPDH	25	9	47	5	Median	Case–control study
Zuo	2017	GC	China	37	qRT-PCR	RNU6B	18	13	19	6	Median	Case–control study

**Abbreviation**: CCA, cholangiocarcinoma; CRC, colorectal cancer; EC, endometrial cancer; PC, pancreatic carcinoma.

**Table 2:** Subgroup analysis of the role of UCA1 in LNM in different types of cancer

Author	Year	Tumor type	Country	Sample size	Detection method	Reference Control	Cut-off value	Survival analysis	Multivariate analysis	HR statistic	Hazard ratios (95%)	Follow-up (months)	Research type of the studies
Bian	2016	CRC	China	90	qRT-PCR	β-Actin	Median	OS	Yes	Data in paper	2.40 (1.04–5.50)	75	Case–control study
Bian	2016	CRC	China	105	qRT-PCR	β-Actin	Median	OS	NO	Survival curve	1.62 (0.90–2.91)	125	Case–control study
Cai Q.	2017	GBC	China	45	qRT-PCR	GAPDH	Median	OS	NO	Survival curve	2.08 (1.01–4.29)	40	Case–control study
Chen D.	2015	PDAC	U.S.A.	63	qRT-PCR	NA	Median	OS	NO	Survival curve	2.76 (1.15–6.61)	21	Case–control study
Chen P.	2016	Pancreatic	China	128	qRT-PCR	GAPDH	Median	OS	Yes	Data in paper	1.50 (1.01–2.24)	60	Case–control study
Fu	2015	PDAC	China	80	qRT-PCR	GAPDH	Median	OS	Yes	Data in paper	2.02 (1.02–4.01)	40	Case–control study
Gao	2015	GC	China	20	qRT-PCR	GAPDH	NA	OS	Yes	Data in paper	2.02 (1.02–3.37)	40	Case–control study
Han	2014	CRC	China	80	qRT-PCR	GAPDH	Mean	OS	NO	Survival curve	7.44 (1.84–30.15)	42.6	Case–control study
He	2017	Glioma	China	80	qRT-PCR	β-Actin	NA	OS	NO	Survival curve	1.52 (0.61–3.78)	35	Case–control study
Jiao	2016	Esophageal	China	66	qRT-PCR	NA	Median	OS	NO	Survival curve	3.36 (1.48–7.61)	30	Case–control study
Johanna	2017	UC	Germany	106	qRT-PCR	SDHA/TBP	Median	OS	Yes	Data in paper	0.57 (0.37–0.90)	200	Case–control study
Khakiani	2017	GC	Iran	40	qRT-PCR	GUSB	Median	OS	NO	Survival curve	4.08 (1.63–10.22)	100	Case–control study
Li	2014	ESCC	China	90	qRT-PCR	GAPDH	Mean	OS	Yes	Data in paper	2.63 (1.42–5.87)	60	Case–control study
Liu	2016	BC	China	54	qRT-PCR	GAPDH	Median	OS	NO	Survival curve	2.08 (1.04–4.15)	60	Case–control study
Lu	2016	EC	China	45	qRT-PCR	GAPDH	Median	OS	NO	Survival curve	3.95 (1.20–12.96)	60	Case–control study
Lu Y.	2017	RCC	China	50	qRT-PCR	GAPDH	Median	OS	NO	Survival curve	3.20 (1.41–7.26)	60	Case–control study
Na	2015	PC	China	40	qRT-PCR	GAPDH	Median	OS	Yes	Survival curve	1.52 (1.23–1.88)	60	Case–control study
Ni	2015	CRC	China	54	qRT-PCR	GAPDH	Median	OS	NO	Survival curve	3.14 (1.17–8.41)	50	Case–control study
Nie	2016	NSCLC	China	112	qRT-PCR	β-Actin	Youden index	OS	Yes	Data in paper	1.41 (1.08–1.84)	60	Case–control study
Qian	2017	HSCC	China	53	qRT-PCR	β-Actin	Median	OS	NO	Survival curve	1.83 (0.89–3.78)	60	Case–control study
Sedlarikova	2017	MM	Czech	64	qRT-PCR	RPLP0	NA	OS	Yes	Data in paper	1.94 (1.17–3.22)	60	Case–control study
Tao	2015	CC	China	80	qRT-PCR	β-Actin	Fourth quartile of the expression level of UCA1.	OS	Yes	Data in paper	2.00 (1.01–3.98)	60	Case–control study
Wang F.	2015	HCC	China	98	qRT-PCR	RNU6B	Median	OS	Yes	Data in paper	1.86 (1.08–3.21)	60	Case–control study
Wang H.	2015	LC	China	60	qRT-PCR	GAPDH	Median	OS	Yes	Data in paper	1.94 (1.06–3.26)	60	Case–control study
Wang Y.	2017	RCC	China	384	qRT-PCR	NA	NA	OS	Yes	Data in paper	1.92 (1.36–2.70)	150	Case–control study
Wen	2017	Osteosarcoma	China	151	qRT-PCR	GAPDH	NA	OS	Yes	Data in paper	2.52 (1.35–4.83)	60	Case–control study
Xu	2017	CCA	China	68	qRT-PCR	GAPDH	NA	OS	Yes	Data in paper	2.27 (1.31–3.94)	60	Case–control study
Yang Y.J.	2016	OC	China	53	qRT-PCR	GAPDH	Median	OS	Yes	Data in paper	6.32 (1.12–35.68)	50	Case–control study
Yang Z.	2015	HCC	Korea	240	qRT-PCR	NA	Median	OS	Yes	Data in paper	1.99 (0.84–4.69)	120	Case–control study
Zhang L.	2016	OC	China	110	qRT-PCR	GAPDH	Median	OS	Yes	Data in paper	1.69 (1.01–2.83)	60	Case–control study
Zhang S.	2017	PC	China	47	qRT-PCR	GAPDH	NA	OS	NO	Survival curve	2.09 (0.80–5.46)	60	Case–control study
Zhao	2017	Glioma	China	64	qRT-PCR	GAPDH	>22.20	OS	NO	Data in paper	7.37 (3.03–17.90)	48	Case–control study
Zheng	2015	GC	China	112	qRT-PCR	RNU6B	Median	OS	Yes	Data in paper	2.35 (1.22–4.52)	60	Case–control study
Zheng Z.	2018	HCC	China	105	qRT-PCR	GAPDH	Median	OS	Yes	Data in paper	3.65 (1.17–4.65)	60	Case–control study
Zhou	2017	PC	China	72	qRT-PCR	GAPDH	Median	OS	NO	Survival curve	1.87 (0.54–6.53)	60	Case–control study
Zuo	2017	GC	China	37	qRT-PCR	RNU6B	Median	OS	Yes	Data in paper	2.92 (1.07–7.96)	40	Case–control study

**Abbreviation**: CCA, cholangiocarcinoma; CRC, colorectal cancer; EC, endometrial cancer; PC, pancreatic carcinoma.

## Meta-analysis results

### Association between UCA1 and LNM

**Figure 2:**
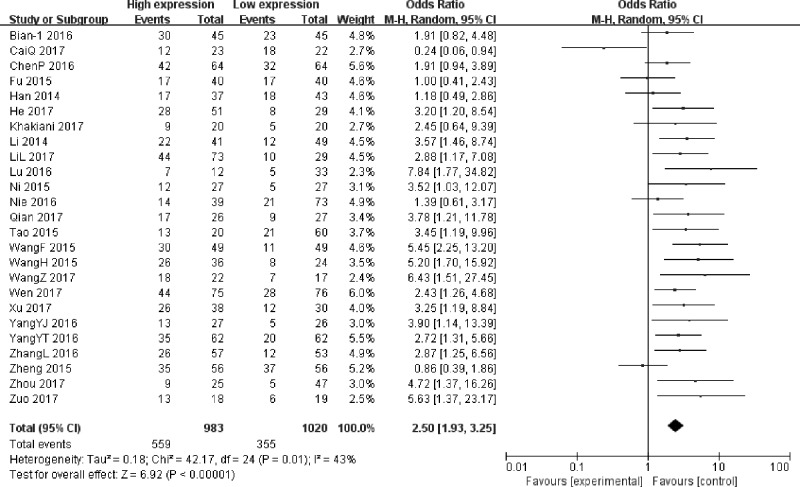
Forest plot for the association between UCA1 expression levels with LNM

The 25 studies (Table 1) reported a total of 2003 patients with LNM based on different UCA1 expression levels. The random-effects model was adopted as the moderate heterogeneity (*I^2^*= 43%, *P*=0.01). Analysis showed that the OR of high UCA1 expression group versus low UCA1 expression group was 2.50 (95% CI: 1.93–3.25; *P*<0.00001) (Figure 2), which revealed that a higher UCA1 expression predicted more LNM. The result indicated that patients with high UCA1 expression in cancer tissues were more susceptible to LNM.

### Association between UCA1 and OS

**Figure 3:**
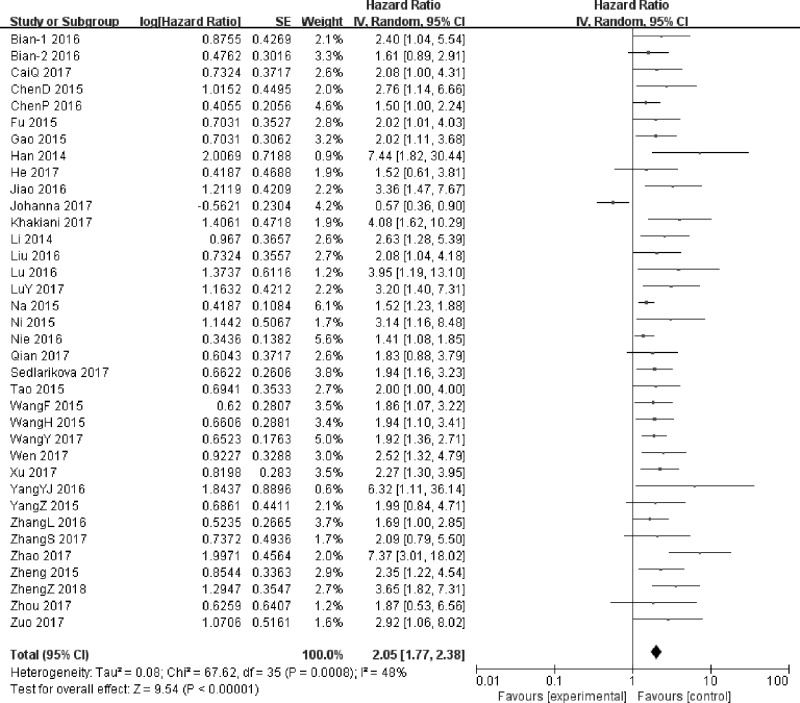
Forest plot for the association between UCA1 expression levels with OS

A total of 36 studies including 3146 patients were assessed for the correlation between UCA1 and OS (Table 2), High UCA1 expression was significantly correlated with poor prognosis, compared with low UCA1 expression in a pooled analysis of all studies (HR = 2.05; 95% CI: 1.77–2.38; *P*<0.00001) (Figure 3). The random-effects model was used because of the moderate heterogeneity (*I^2^* = 48%, *P*=0.0008). In other words, high UCA1 expression group shortened the OS compared with low UCA1 expression group.

### Subgroup analysis

Subgroup analyses across several different variables were further performed to investigate the heterogeneity of the studies for meta-analysis of UCA1 and LNM or OS. The LNM-related data were stratified into subgroups based on sample size, tumor type, cut-off value and reference control. The assessment results in each subgroup are also shown in Table 3. Subgroup analysis by sample size explored that high UCA1 expression status was related to high LNM numbers both in big (*n*≥100, OR = 1.99, 95% CI: 1.50–2.65, *P*<0.0001) and small sample size group (*n*<100, OR = 2.71, 95% CI: 2.12–3.47, *P*<0.00001). And we also found a significantly positive correlation between UCA1 expression and LNM when grouped by different cut-off value [By median (OR = 2.48, 95% CI: 1.63–3.78, *P*<0.0001) and By others (OR = 2.53, 95% CI: 1.92–3.34, *P*<0.00001)]. However, when conducting subgroup analyses on tumor type, we found no significant correlation between high UCA1 expression and LNM among the studies in respiratory system (OR = 2.54, 95% CI: 0.70–9.23, *P*=0.16). According to the results presented in Table 3, when divided by reference control, the subgroup analysis showed that up-regulated UCA1 was associated with more LNM in GAPDH group (OR = 2.41, 95% CI: 1.91–3.04, *P*<0.00001) and β-actin group (OR = 2.35, 95% CI: 1.54–3.57, *P*<0.0001), while no significant association in RNU6B/GUSB group (OR = 2.69, 95% CI: 0.95–7.56, *P*=0.06).

**Table 3:** Subgroup analysis of the role of UCA1 in LNM in different types of cancer

Subgroup analysis	No. of studies	No. of patients	Test of relationship		Test of heterogeneity	
			HR (95% CI)	*P*-value	I^2^ (%)	Q-value
Overall	25	2003	2.50 (1.93–3.25)	<0.00001	43	0.01
Sample size						
<100	18	1164	2.71 (2.12–3.47)	<0.00001	46	0.02
≥100	7	839	1.99 (1.50–2.65)	<0.00001	23	0.25
Tumor type						
Respiratory system	2	172	2.54 (0.70–9.23)	0.16	71	0.06
Digestive system	17	1320	2.27 (1.61–3.20)	<0.00001	52	0.006
Reproductive system	3	208	3.65 (1.96–6.81)	<0.0001	0	0.51
Others	3	303	2.90 (1.77–4.77)	<0.0001	0	0.63
Cut off						
Median	15	1077	2.48 (1.63–3.78)	<0.0001	59	0.002
Others	10	926	2.53 (1.92–3.34)	<0.00001	0	0.54
Reference control						
GAPDH	16	1301	2.41 (1.91–3.04)	<0.00001	45	0.03
β-Actin	5	415	2.35 (1.54–3.57)	<0.0001	0	0.5
RNU6B/GUSB	4	287	2.69 (0.95–7.56)	0.06	74	0.009

The OS-related data were stratified into subgroups based on sample size, tumor type, cut-off value, follow-up time, analysis method, race and reference control. The detailed assessment results in each subgroup are also shown in Table 4. Subgroup analysis by sample size, cut-off value, follow-up time, analysis method and reference control all revealed that high UCA1 expression was significantly associated with poor OS in each groups. However, when conducting subgroup analyses on tumor type, we found high UCA1 expression was remarkably related to poor OS among respiratory system, digestive system, reproductive system and other systems but no significant correlation between high UCA1 expression and OS among the studies in urinary system (HR = 1.54, 95% CI: 0.98–2.40, *P*=0.06). As for different race for UCA1, the relationship between UCA1 expression and OS was significant in Asian group (HR = 1.90, 95% CI: 1.72–2.10, *P*<0.00001), but not in others group (HR = 1.39, 95% CI: 0.52–3.71, *P* = 0.51).

**Table 4:** Subgroup analysis of the role of UCA1 in OS in different types of cancer

Subgroup analysis	No. of studies	No. of patients	Test of relationship		Test of heterogeneity	
			HR (95% CI)	*P*-value	I^2^ (%)	Q-value
Overall	36	3146	2.05 (1.77–2.38)	<0.00001	48	0.0008
Sample size						
<100	26	1593	2.03 (1.80–2.30)	<0.00001	14	0.26
≥100	10	1553	1.67 (1.25–2.22)	0.0005	71	0.0003
Tumor type						
Respiratory system	2	172	1.50 (1.17–1.91)	0.001	0	0.32
Digestive system	20	1654	2.18 (1.87–2.55)	<0.00001	0	0.76
Urinary system	6	699	1.54 (0.98–2.40)	0.06	78	0.0003
Reproductive system	3	208	2.10 (1.32–3.33)	0.002	39	0.19
others system	5	413	2.37 (1.75–3.22)	<0.00001	49	0.10
Region						
Asian	33	2913	1.90 (1.72–2.10)	<0.00001	21	0.14
Non Asian	3	233	1.39 (0.52–3.71)	0.51	88	0.0002
Cut off						
Median	24	1906	2.01 (1.65–2.45)	<0.00001	51	0.0002
Others	12	1240	1.92 (1.65–2.24)	<0.00001	43	0.06
Analysis method						
Non-multivariable analysis	15	918	2.55 (2.04–3.18)	<0.00001	9	0.35
Multivariable analysis	21	2228	1.83 (1.55–2.16)	<0.00001	51	0.004
Reference control						
GAPDH	19	1416	1.96 (1.72–2.22)	<0.00001	35	0.06
β-actin	6	520	1.57 (1.27–1.93)	<0.0001	0	0.81
Other controls	10	1210	2.00 (1.37–2.93)	0.0004	72	0.0002
Follow-up (months)						
<60	11	642	2.71 (2.09–3.51)	<0.00001	14	0.31
≥60	25	2504	1.71 (1.54–1.89)	<0.00001	47	0.005

### Sensitivity analysis

**Figure 4:**
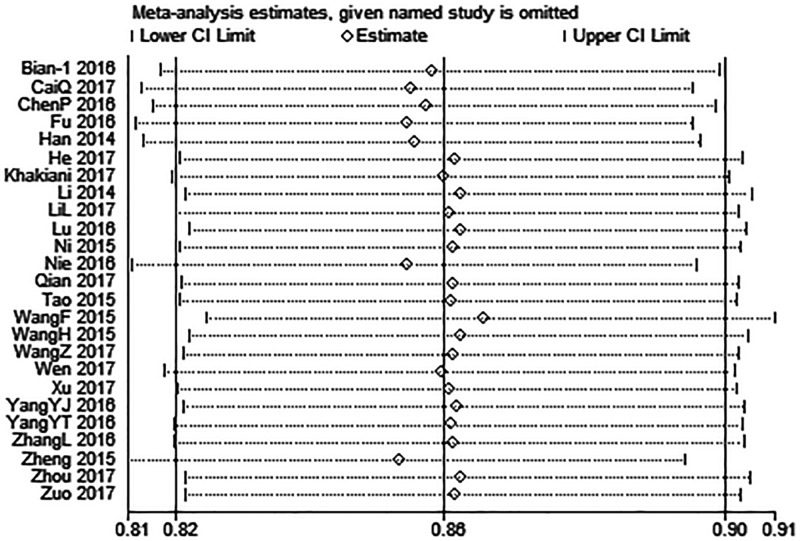
Sensitivity analysis for the association between UCA1 expression levels with LNM

**Figure 5:**
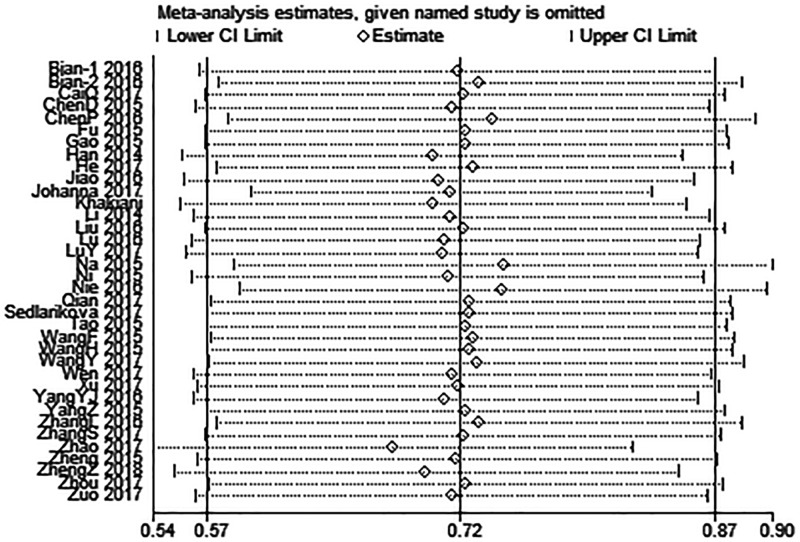
Sensitivity analysis for the association between UCA1 expression levels with OS

Multiple sensitivity analyses were carried out to evaluate whether individual study influenced pooled ORs or HRs by excluding one study by turns. It was found that none of the exclusions of a specific study would change the magnitude or direction of the summary effect for the correlation between UCA1 expression and LNM or OS, which further confirmed the validity of the results (Figures 4 and 5).

### Publication bias

**Figure 6:**
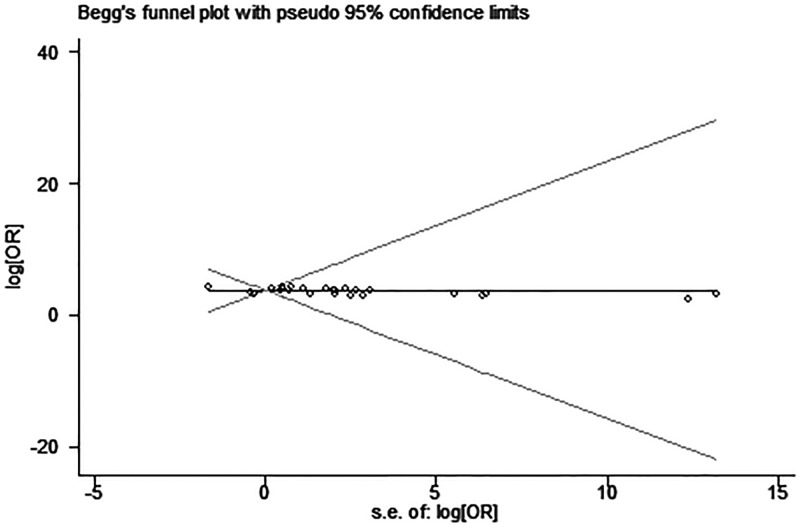
Funnel plot analysis of potential publication bias for LNM

**Figure 7:**
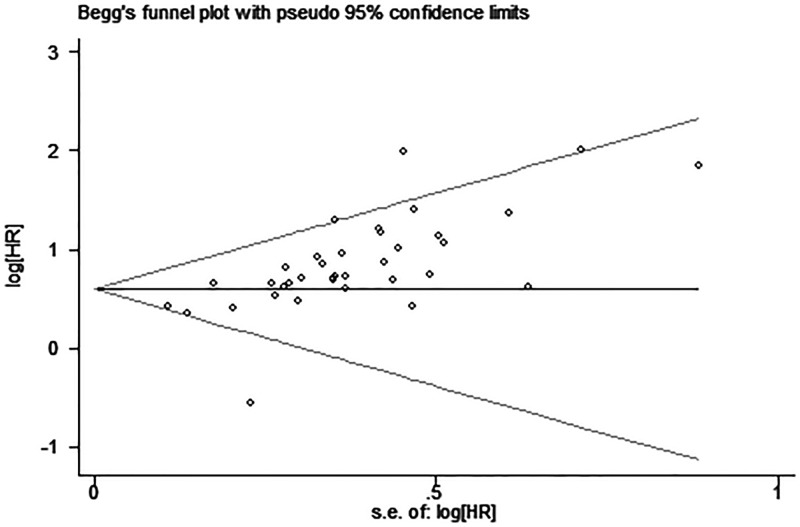
Funnel plot analysis of potential publication bias for OS

**Figure 8:**
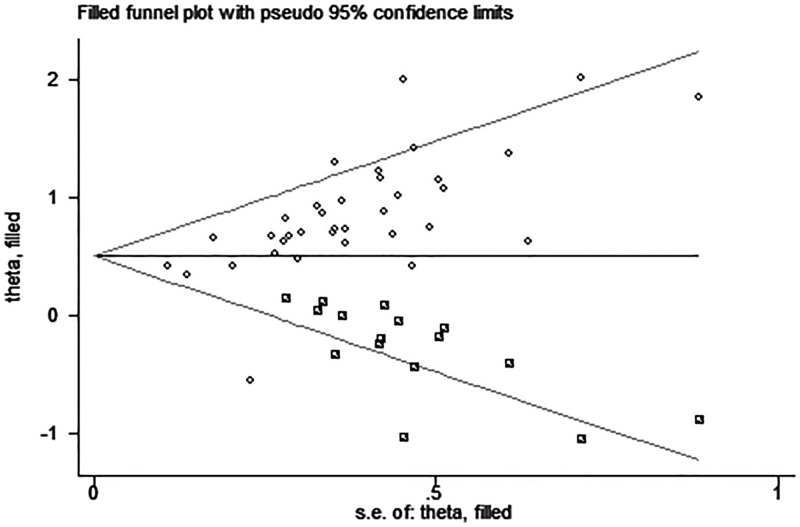
Funnel plot analysis of potential publication bias for OS with trim and fill

Egger’s test and funnel plot were introduced to evaluate potential publication bias in our present meta-analysis. No evidence supporting publication bias was found in analysis between UCA1 and LNM (Egger’s test, *t* = 1.31, *P*=0.202) (Figure 6). However, the shapes of funnel plot were asymmetric and Egger’s test displayed slightly publication bias for the HR evaluation of OS (Egger’s test, *t* = 4.76, *P*<0.05) (Figure 7). Because of this, trim and fill was used to perform a sensitivity analysis. This method conservatively conjectures hypothetical negative unpublished studies to reflect positive studies that lead to funnel plot asymmetry, and then a symmetrical funnel plot appears (Figure 8). While the statistically significant relationship between UCA1 expression and OS was also shown in pooled analysis incorporating the hypothetical studies, indicating that the result was stable and publication did not have an impact on it though publication bias exists.

## Discussion

Evidence from multiple publications demonstrated that lncRNAs, similar to protein-coding genes, can act as oncogenes or tumor suppressor genes, which involving in a variety of tumorigenesis processes, including proliferation, invasion, migration and apoptosis. With the rapid development of high-throughput genome-wide analysis technology, more and more functional lncRNAs have been found to have potential value on predicting cancer progression. UCA1, a novel functional lncRNA which was first discovered in 2006 in human bladder cancer. In recent years, a growing number of studies have shown that UCA1 up-regulated in several cancers, including HCC, GC and lung cancer [7]. UCA1 involved in tumor proliferation, invasion, migration and apoptosis, and played an important role in tumor progression, metastasis and prognosis. However, a persuasive support of the UCA1 in clinical practice is still controversial, partially due to the uncertainty of the relationship between UCA1 and metastasis or prognosis implication. Several literatures established a statistically significant relationship between high UCA1 expression and lymph node metastasis or prognosis. Nevertheless, some studies showed no statistical impact of UCA1 dysregulation on cancer metastasis and prognosis. In order to combine previous research results about UCA1 and cancers to arrive at a summary conclusion, a comprehensive study is performed.

In the present meta-analysis, we systematically explore the relationship between UCA1 and cancer metastasis or prognosis. The results of the current study demonstrated that high UCA1 expression level was positively related to increasing the risk of LNM in cancer patients. Moreover, we also identified that there was a significantly positive correlation between high UCA1 expression and short OS in cancer patients. In multiple sensitivity analyses, we did not detect any substantial difference in pooled estimates, and there was no excessive influence on the overall results in any individual study.

The exact mechanisms underlying the association between elevated UCA1 expression and more LNM or poor prognosis is poorly understood, and the related reports are not the same, but many similarities were still existed. Several literatures have suggested potential mechanisms that could be involved in the metastasis and prognostic impact of UCA1 on carcinogenesis. First, UCA1 could act as a key competing endogenous RNA (ceRNA) or sponge for *miR-204-5p, miR-193a-3p, miR-145, miR-143, miR-216b, miR-203, miR-196a-5p* and *miR-135a* in several different cancers. For example, Zhang et al. found that UCA1 could directly interacted with *miR-204* and functioned as a ceRNA, thus regulating the expression of ATF2 and promoting cell proliferation and metastasis in prostate cancer [16]. Nie et al. found UCA1 up-regulated the expression level of *miR-193a-3p* target gene ERBB4 by competitively ‘spongeing’ *miR-193a-3p* in NSCLC [26]. In bladder cancer, Xue et al. demonstrated that UCA1 induced epithelial–mesenchymal transition (EMT) of bladder cancer cells through up-regulating the expression of zinc finger E-box binding homeobox 1 and 2 (ZEB1 and ZEB2), and also regulated cell migration and invasion of bladder cancer by suppressing *miR-145* and its target gene the actin-binding protein fascin homologue 1 (FSCN1) [48]. In HCC, Wang et al. found UCA1 acted as an endogenous sponge through binding to *miR-216b* directly and down-regulated the expression of *miR-216b*. UCA1 reversed the inhibitory effect on the growth and metastasis of *miR-216b* of HCC, which might be involved in the suppression of fibroblast growth factor receptor 1 (FGFR1) expression, a target gene of *miR-216b*, and the activation of ERK signaling pathway [42]. In addition, Xiao et al. showed that UCA1 up-regulation promoted cell EMT in HCC via sponging to *miR-203* effectively and thus activating the expression of transcription factor Snail2 and promote HCC progression [49]. In bladder cancer, UCA1 could promote glycolysis by up-regulating hexokinase 2 through both activation of STAT3 and repression of *miR-143* [50]. UCA1-activated transcription factor CREB which resulting in *miR-196a-5p* expression by binding with its promoter and thereby modulating the influence on cisplatin/gemcitabine resistance [51]. Second, UCA1 promoted the progression of different cancer by activating of the Wnt/β-catenin signaling pathway. UCA1 down-regulation increased the tamoxifen sensitivity through inhibiting Wnt/β-catenin pathway in breast cancer cells while UCA1 up-regulation promoted EMT of breast cancer cells by activating Wnt/β-Catenin signaling pathway [31,52]. Silence UCA1 suppressed cell proliferation and metastasis and induced cell apoptosis of oral squamous cell carcinoma, which might be significantly correlated with the activation level of the Wnt/β-Catenin signaling pathway [11]. Fan et al. indicated that UCA1 could increase the cisplatin resistance of bladder cancer cells by regulating the Wnt signaling [53]. Third, UCA1 overexpression could promote cancer metastasis by activation of metastasis-related genes including GRK2/ERK-MMP9, EZH2/AKT, p21/E-cadherin, iASPP, KLF4-KRT6/13, FGFR1/ERK and ZEB1/2-FSCN1. UCA1 overexpression could increase the metastatic ability of GC cells through regulating GRK2 protein stability by promoting Cbl-c-mediated GRK2 ubiquitination and degradation, thus activate the ERK-MMP9 signaling pathway [54]. Mechanically, UCA1 promoted the cell proliferation and metastasis of GBC by recruiting enhancer of zeste homolog 2 (EZH2) to the promoter of p21 and E-cadherin, and epigenetically suppressing their transcript [43]. He et al. demonstrated that UCA1 overexpression promoted cell proliferation and migration of glioma, to regulate the tumor growth and metastasis via *miR-182* dependent iASPP regulation [35]. Wang et al. suggested that UCA1 overexpression contributed to the growth and metastasis of HCC via inhibiting *miR-216b* and activating FGFR1/ERK signaling pathway [42]. Simultaneously, UCA1 also remarkably associated with prognosis of patients with different cancer and may be a potential diagnosis biomarker in hepatocellular cancer, CRC and GC.

Otherwise, some limitations to this meta-analysis should be taken into account. First, the cut-off values of UCA1 high and low expression were lack of uniform standard due to different methods and criteria in different types of cancer, which may result in some heterogeneity and affect the results of the study. Second, most studies tended to report positive results rather than negative results; our meta-analysis may overestimate the significance of UCA1 to some extent. Third, some of the HRs were estimated from survival curves rather than directly obtained from the primary studies. Lastly, most of the included studies were performed in the population from Asian countries rather than worldwide population; our results should be substantiated by additional studies in other races. Although there are some limitations, but this current meta-analysis still has its noteworthy advantages. First, 38 literatures which including a total of 3411 cases and 19 types of cancer were included in this meta-analysis. The sample size included was the largest, which significantly improved the statistical efficiency and accuracy of the test. Second, the number of search databases were greater and cancer types were more comprehensive in this meta-analysis compared with previous reports. Finally, the inclusion and exclusion criteria were more stringent and the quality of the literatures incorporated was higher.

## Conclusion

In conclusion, even some limitations mentioned above, our meta-analysis reveals that the expression level of UCA1 was significantly associated with metastasis and prognosis in different types of cancer. The higher expression of UCA1, the higher probability of occurrence of LNM cancer patients suffer with. Meanwhile, shorter OS may be observed in the patients with high UCA1 expression. Thus, UCA1 might be a novel predictive marker for estimating the metastasis and prognosis in different types of cancer. However, the significance of UCA1 in LNM in respiratory system cancers and RNU6B/GUSB reference control group should incorporate more studies to validate this result, and so does in urinary system prognosis and non-Asian people prognosis.

## Abbreviations

BC: bladder cancer
CCA: cholangiocarcinoma
ceRNA: competing endogenous RNA
CI: confidence interval
CRC: colorectal cancer
CREB: cAMP response element-binding protein
EC: endometrial cancer
EMT: epithelial–mesenchymal transition
EZH2: enhancer of zeste homolog 2
FGFR1: fibroblast growth factor receptor 1
FSCN1: fascin homologue 1
GBC: gallbladder cancer
GC: gastric cancer
HCC: hepatocellular carcinoma
HR: hazard ratio
iASPP: inhibitor of apoptosis-stimulating protein of p53
lncRNA: long noncoding RNA
LNM: lymph node metastasis
MM: multiple myeloma
NOS: Newcastle–Ottawa Scale
NSCLC: non-small-cell lung cancer
OC: ovarian cancer
OR: odd ratio
OS: overall survival
PC: pancreatic carcinoma
PDAC: pancreatic ductal adenocarcinoma
qRT-PCR: quantitative real-time PCR
RCC: renal cell carcinoma
STAT3: phospho-signal transducer and transcription activator 3
UC: urothelial carcinoma
UCA1: urothelial carcinoma associated 1
